# Clinical outcomes of a remimazolam-based sedation regimen in patients receiving ECMO: a retrospective comparative study

**DOI:** 10.3389/fmed.2026.1819593

**Published:** 2026-06-08

**Authors:** Dalong Zhang, Ying Liu, Tongrui Zhang, Yuanjie Shang, Huijun Dong, Xingguo Niu

**Affiliations:** Department of Critical Care Medicine, The Fifth Clinical Medical College of Henan University of Chinese Medicine (Zhengzhou People's Hospital), Zhengzhou, Henan, China

**Keywords:** delirium, drug combination, ECMO, injectable remimazolam benzenesulfonate, retrospective study, sedatives

## Abstract

**Objective:**

Recent meta-analyses have established the overall safety of remimazolam in general surgical populations, but evidence in high-risk subgroups such as patients requiring extracorporeal membrane oxygenation (ECMO) remains absent. This study aimed to compare the sedative effects of remimazolam-based vs. midazolam-based sedation regimens in adults requiring ECMO, with a focus on the incidence of delirium.

**Methods:**

This single-center retrospective study included patients receiving ECMO from January 2023 to October 2025. The primary analysis cohort comprised patients receiving veno-arterial ECMO (VA-ECMO, *n* = 44), divided into a remimazolam group (Group R*, n* = 22) or a midazolam group (Group M, *n* = 22). The primary outcome was delirium incidence assessed by the Confusion Assessment Method for the Intensive Care Unit (CAM-ICU).

**Results:**

For the primary outcome, delirium occurred in 0/22 (0%) patients in Group R vs. 6/22 (27.3%) in Group M (risk difference −27.3%, 95% CI −45.9% to −8.7%, *p* = 0.021 by Fisher's exact test). Due to zero events in Group R, Firth's penalized likelihood regression adjusting for Acute Physiology and Chronic Health Evaluation II (APACHE-II) yielded an odds ratio of 0.06 (95% CI 0.00 to 0.56, *p* = 0.009), and multivariable and propensity score adjustments produced consistent results. E-value analysis (point estimate 2.0, lower CI bound 1.3) indicated that a relatively modest unmeasured confounder could potentially shift the confidence interval to include the null. For secondary outcomes, Group R had shorter recovery time after decannulation (24.90 ± 3.36 h vs. 29.84 ± 4.53 h, *p* < 0.001), better-preserved muscle strength (median MRC grade 1 vs. 0, *p* < 0.001), and lower incidences of hypotension and bradycardia (both *p* < 0.05).

**Conclusion:**

In this retrospective exploratory study, a remimazolam-based sedation regimen was associated with lower delirium incidence, faster recovery, and better-preserved muscle strength compared with a midazolam-based regimen. These findings reflect a comparison of two distinct sedation regimens rather than a head-to-head drug comparison of remimazolam vs. midazolam. These hypothesis-generating findings warrant further validation in prospective studies.

## 1 Introduction

In recent years, ECMO, as an advanced life support technology, has been increasingly utilized in the treatment of critically ill patients. ECMO is primarily indicated for patients with severe cardiopulmonary failure and provides continuous extracorporeal respiratory and circulatory support, allowing the heart and lungs to rest and recover (1–3). ECMO can be classified into two types: VA-ECMO, which provides both cardiac and respiratory support, and veno-venous ECMO (VV-ECMO), which provides respiratory support alone.

Sedation is a crucial component of adjunctive therapy in ECMO. Appropriate sedation not only alleviates patient anxiety and agitation, thereby mitigating the stress response, but also improves patient-device interaction, ensuring the efficacy and safety of mechanical ventilation and other life-support interventions (2, 4–6). Proper sedation can prevent the exacerbation of cardiac insufficiency and reduce postoperative stress and hemodynamic complications. Commonly used sedative agents include propofol, midazolam, and dexmedetomidine, however, no single agent has yet been established as the definitive standard of care.

Recently, remimazolam benzenesulfonate, an ultrashort-acting injectable sedative, has been introduced for procedural sedation and general anesthesia. This agent is characterized by rapid onset, brief and predictable sedation duration, swift recovery, minimal respiratory and hemodynamic disturbances, and a predictable pharmacological profile (7). Additionally, its compatibility with other pharmacological agents makes it a promising option in clinical sedation practice.

Recent meta-analyses have reported the safety of remimazolam in surgical populations (8, 9). However, these analyses systematically excluded critically ill patients requiring ECMO support. ECMO patients represent a distinct population with altered pharmacokinetics due to hemodilution, drug sequestration in the circuit, and organ dysfunction, which may significantly influence sedative drug behavior and delirium risk. To date, no study has specifically investigated remimazolam-based sedation in ECMO patients.

Therefore, this study aimed to assess the clinical profile, particularly the sedative effects and delirium incidence, of a remimazolam-based sedation regimen compared to a midazolam-based regimen in critically ill patients requiring ECMO. Recognizing the fundamental differences between VA-ECMO and VV-ECMO, we designated the VA-ECMO population as the primary cohort for analysis. The primary objective was to compare the incidence of delirium between the two regimens within this primary cohort. Secondary objectives included comparisons of recovery time after decannulation, muscle strength, time to achieve sedation target, sedation quality, and the incidence of adverse effects. We hypothesized that, in this unique and understudied population, the remimazolam-based regimen would be associated with a lower incidence of delirium compared to the midazolam-based regimen, providing the first exploratory data to guide future research.

## 2 Methods

### 2.1 Study design and participants

This was a single-center, retrospective cohort study conducted at the Fifth Clinical Medical College of Henan University of Chinese Medicine (Zhengzhou People's Hospital). A retrospective screening was conducted on all adult patients (aged ≥18 years) who received VA-ECMO or VV-ECMO support at our institution from January 2023 to October 2025.

**Figure 1:**
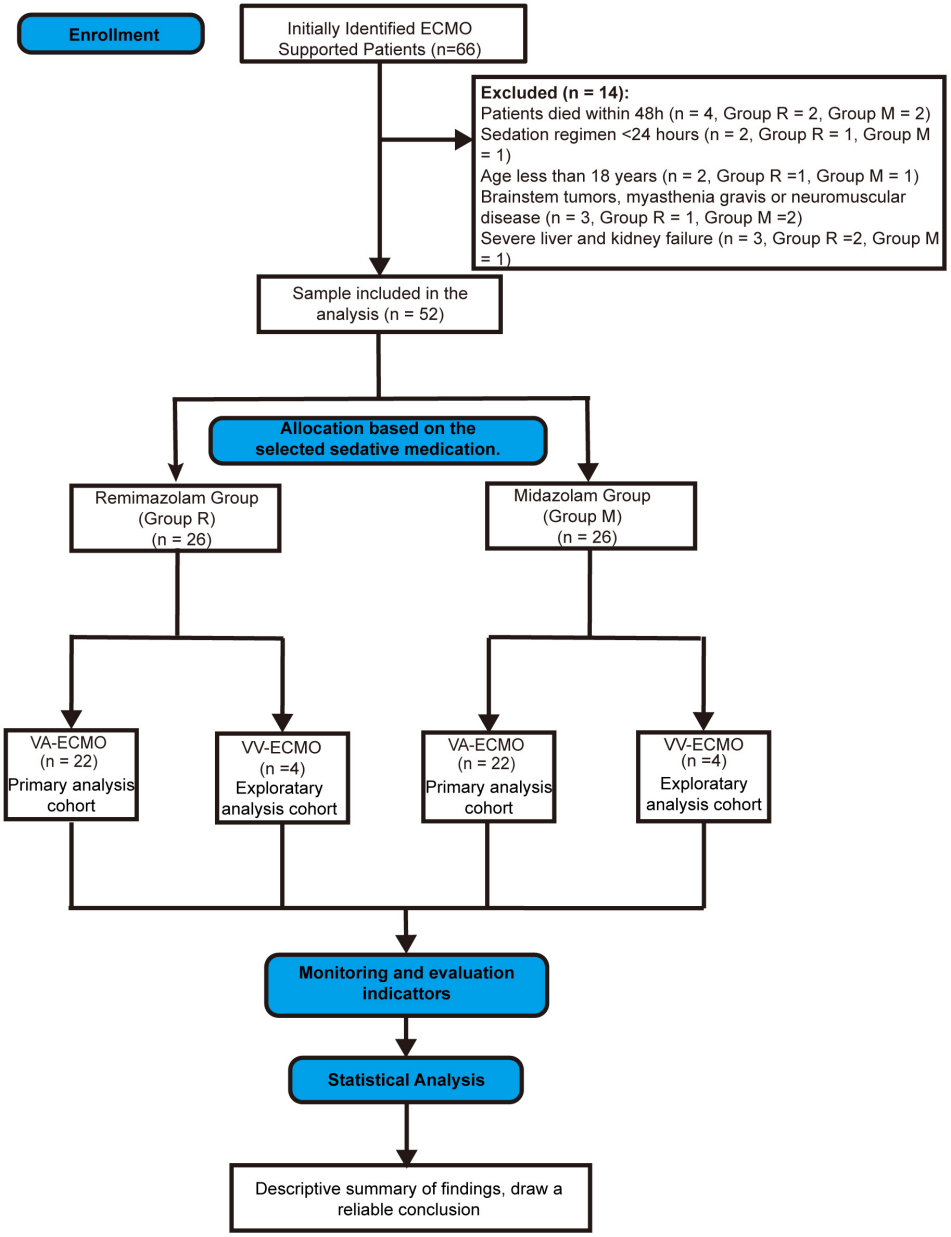
Flowchart of the study. Flow diagram showing patient screening, exclusion, allocation, and analysis cohorts. Of 66 initially screened patients receiving ECMO support, 14 were excluded, and 52 were included in the analysis. Patients were divided based on the primary sedative into a remimazolam group (Group R, *n* = 26) or a midazolam group (Group M, *n* = 26). The primary analysis cohort comprised patients receiving VA-ECMO (*n* = 22 per group), and the exploratory analysis cohort comprised patients receiving VV-ECMO (*n* = 4 per group).

During the study period, 66 patients received ECMO support at our institution. After applying inclusion and exclusion criteria, 52 patients were included in the analysis. The primary analysis cohort consisted of patients who received VA-ECMO support (*n* = 44), including 22 patients in the remimazolam group (Group R) and 22 patients in the midazolam group (Group M). Patients who received VV-ECMO (*n* = 8, 4 per group) were analyzed separately and presented in the Supplementary material as an exploratory cohort (Figure 1).

This study was reported following the Strengthening the Reporting of Observational Studies in Epidemiology (STROBE) guideline. The STROBE checklist is provided as Supplementary material.

### 2.2 Inclusion and exclusion criteria

The inclusion criteria were as follows: (1) adult patients who met the indications for ECMO therapy and required sedation during ECMO support; and (2) patients who received ECMO support; and (3) patients who received one of the two predefined primary sedative regimens (remimazolam or midazolam) for at least 24 h.

The exclusion criteria were as follows: (1) died within 48 h; (2) were under 18 years of age; (3) had brainstem tumors, myasthenia gravis, or neuromuscular disease; (4) had severe liver failure (defined as Child-Pugh Class C) or kidney failure (defined as an estimated glomerular filtration rate < 15 mL/min/1.73 m2 or requiring renal replacement therapy prior to ECMO).

Patients who died within 48 h or received the predefined sedation for less than 24 h were excluded to ensure sufficient exposure time to accurately assess the sedative efficacy and the incidence of delirium.

### 2.3 ECMO management

All patients were managed by a dedicated, multidisciplinary ECMO team in accordance with institutional protocols. The same ECMO system was used for all patients (Sorin SCP console with integrated centrifugal pump and Xenios AG hilite 7000LT oxygenator) to minimize variability in circuit characteristics and potential drug adsorption. The femoral vein-internal jugular vein configuration was used for VV-ECMO, whereas the femoral artery-femoral vein configuration was used for VA-ECMO.

Standardized operational procedures included: initial ECMO blood flow titrated to achieve target mixed venous oxygen saturation (SvO_2_) >70% for VA-ECMO or adequate arterial oxygenation (PaO_2_ >60 mmHg) for VV-ECMO; sweep gas flow adjusted to maintain PaCO_2_ between 35 and 45 mmHg; systemic anticoagulation with unfractionated heparin targeting activated clotting time (ACT) of 180–220 s or activated partial thromboplastin time (APTT) of 1.5–2.0 times baseline; and daily surveillance for circuit complications.

### 2.4 Analgesic sedation programs

During the study period, patients received one of two analgesic sedation strategies, selected at the discretion of the attending intensivist based on clinical judgment and drug availability. All patients received concomitant analgesia with remifentanil and adjuvant sedation with dexmedetomidine. The specific dosing regimens are detailed in Table 1.

**Table 1:** Patient analgesia and sedation management program.

Item	Group R	Group M
Primary sedatives	Injectable remimazolam benzenesulfonate	Midazolam
Dosage	0.1–0.3 mg·kg^−1^·h^−1^	0.1–0.2 mg·kg^−1^·h^−1^
Adjuvant sedative	Dexmedetomidine	Dexmedetomidine
Dosage	0.1–0.5 μg·kg^−1^·h^−1^	0.1–0.5 μg·kg^−1^·h^−1^
Analgesic	Remifentanil	Remifentanil
Dosage	0.025–0.1 μg·kg^−1^·min^−1^	0.025–0.1 μg·kg^−1^·min^−1^
Rescue sedative	Propofol (as needed)	Not routinely used
Indication/Dosage	For invasive procedures or inadequate sedation, 0.1–0.6 mg/kg	

In Group R, propofol was administered as a temporary rescue bolus (0.1–0.6 mg/kg) only during invasive procedures or when transient inadequate sedation occurred. It was not used for continuous maintenance sedation in any patient. This was not routinely used in Group M.

The goal for all patients was to maintain Richmond Agitation and Sedation Scale (RASS) score at −4 to −3 (deep to moderate sedation), the standardized target for ECMO patients in our Intensive Care Unit (ICU). All drug infusions were initiated at the lower end of the dosage range and were dynamically titrated based on continuous assessment of sedation depth, hemodynamic stability, and patient response.

During the study period, remimazolam was introduced into our ICU formulary in June 2024. Prior to this date, all ECMO patients received midazolam as the primary sedative. After June 2024, remimazolam became the preferred sedative, although midazolam remained available for specific clinical situations (e.g., patients with severe hepatic impairment). Consequently, the two groups were not enrolled concurrently: all patients in Group M were treated before June 2024, and all patients in Group R were treated after June 2024. This temporal trend itself may introduce treatment-era bias, which we address in the statistical analysis and acknowledge in the limitations.

### 2.5 Data collection

All data were extracted from the hospital's electronic medical records system. Collected data included demographic characteristics [age, sex, body mass index (BMI)], APACHE-II score at ICU admission, laboratory parameters after ECMO cannulation [white blood cell count, platelet count, total bilirubin, albumin, serum creatinine, plasma plasminogen time (PT), APTT, and the international normalized ratio (INR)], primary etiology for ECMO initiation (cardiac arrest, cardiogenic shock, respiratory failure), key comorbidities (hypertension, diabetes mellitus, coronary artery disease), and vital signs at ECMO initiation.

### 2.6 Outcome definitions

#### 2.6.1 Primary outcome

The primary outcome was the incidence of delirium during the ECMO support period. Delirium was screened daily using the CAM-ICU (10). Assessments were conducted when sedation was lightened to a RASS score ≥ −3, in accordance with our unit's protocol for delirium monitoring in deeply sedated patients.

#### 2.6.2 Secondary outcome

Secondary outcomes included sedation quality, sedation indicators, and adverse events. Sedation quality was assessed by RASS scores at predefined time points (11): before initiation of the study sedation regimen (T0), and at 6 h (T1), 12 h (T2), 24 h (T3), 3 days (T4), 5 days (T5), 7 days (T6), and 9 days (T7) after regimen initiation.

Sedation indicators included muscle strength, time to achieve sedation target (in minutes), recovery time after decannulation (in minutes), and clinical course parameters. To ensure precise measurement, the patient's responsiveness was assessed at 5-min intervals immediately following decannulation until the awakening criterion was met. Muscle strength was evaluated using the MRC grading scale, assessed after ECMO decannulation once the patient was awake and cooperative, typically within 24 h after decannulation. Time to achieve sedation target was defined as the time required to first achieve the target RASS score (−4 to −3) after starting sedation. Recovery time after decannulation was defined as the interval (in hours) from the discontinuation of ECMO support (decannulation) to the time when the patient first demonstrated consistent ability to follow simple verbal commands (e.g., “open eyes,” “squeeze hand”). Clinical course parameters include total ECMO duration (days) and time spent in the ICU (days).

Adverse events occurring during the ECMO support period were systematically captured, including hypotension (defined as a decrease in systolic blood pressure by ≥20% from baseline or to ≤ 80 mmHg), bradycardia (heart rate < 40 beats per minute or a reduction of ≥20% from baseline sustained for ≥30 s), respiratory depression (respiratory rate < 8 breaths per minute and/or peripheral oxygen saturation < 90% in the absence of acute pulmonary pathology), nausea and vomiting (documented episodes recorded in the medical record), and injection site pain (assessed through observation of behavioral responses during drug administration and, when feasible, patient recall after awakening).

#### 2.6.3 Assessment opportunity analysis and adjustment indicators

To assess the potential imbalance in assessment opportunities due to differences in sedation stability between groups, we recorded the number of CAM-ICU-assessable patient-days for each patient. Assessable patient-days were defined as the sum of days during ECMO support on which the patient had at least one documented RASS score ≥ −3. To adjust for differential assessment opportunities, we calculated delirium density. Delirium density was defined as CAM-ICU positive days per 100 assessable patient-days. It was calculated as (total CAM-ICU positive days divided by total assessable patient-days) multiplied by 100.

### 2.7 Statistical analysis

All the statistical analyses were performed using R software (version 4.5.1, R Foundation for Statistical Computing, Vienna, Austria) within the RStudio integrated development environment. The pre-specified primary analysis was a comparison of outcomes between Group R and Group M within the VA-ECMO cohort (*n* = 44). Data description and between-group comparison methods.

For continuous variables, normality was assessed using the Shapiro-Wilk test. Normally distributed measures are expressed as mean ± standard deviation (SD) and compared using independent samples *t*-test. Non-normally distributed measures are expressed as median with interquartile range [IQR] and compared using Mann-Whitney U test. Categorical variables are expressed as number (percentage) and compared using χ^2^ test or Fisher's exact test when expected counts were < 5. These methods were applied to all secondary outcome comparisons.

#### 2.7.1 Primary outcome analysis

For the primary outcome of delirium incidence, between-group comparison was performed using Fisher's exact test due to expected cell counts < 5. The risk difference with 95% confidence interval was calculated using the standard formula for difference between two proportions. The number needed to treat (NNT) was calculated as the reciprocal of the absolute risk reduction, with its 95% confidence interval derived from the confidence limits of the risk difference.

#### 2.7.2 Firth regression and E-value analysis

Due to the complete separation of data (zero events in Group R), conventional logistic regression could not provide reliable odds ratio estimates. Therefore, we performed Firth's penalized likelihood logistic regression, which provides bias-reduced estimates for rare events or separated data (12, 13). We constructed a model adjusting for APACHE-II score, reporting the odds ratio (OR) with 95% confidence interval.

To assess the robustness of the observed association to potential unmeasured confounding, we calculated the E-value (14, 15). The E-value represents the minimum strength of association that an unmeasured confounder would need to have with both treatment and outcome (on the risk ratio scale) to fully explain away the observed association. Due to zero events in the remimazolam group, the E-value was calculated based on the risk difference using a conservative approximation formula: E = 1 + |RD| × 3.5.

#### 2.7.3 Analysis for confounding by indication

To control for confounding by indication and treatment-era bias, we performed multivariable-adjusted and propensity score-adjusted sensitivity analyses. Based on clinical judgment and literature review, prespecified covariates included APACHE-II score, age, sex, and etiology of ECMO initiation (cardiac arrest, cardiogenic shock, respiratory failure). For multivariable adjustment, we incorporated the above covariates individually and jointly into the Firth regression models. For propensity score adjustment, we first calculated a propensity score for each patient using logistic regression with group assignment as the dependent variable and the above covariates as independent variables, the propensity score was then included as a covariate in the Firth regression model. All sensitivity analysis results are presented as a forest plot.

#### 2.7.4 Assessment opportunity analysis and adjustment

To compare the assessment opportunity indicator between groups, the Mann-Whitney U-test was used for the number of assessable patient-days. To adjust for differential assessment opportunities, delirium density was compared between groups using the Poisson exact test.

#### 2.7.5 Sample size and missing data

This was a retrospective exploratory study, and no formal a priori sample size calculation was performed. The sample size was determined by the number of eligible patients during the study period.

Due to the exploratory nature of this study, we did not adjust for multiple comparisons, and findings should be interpreted as hypothesis-generating.

No imputation was performed for missing data, as all key variables were available in the electronic medical records.

## 3 Results

### 3.1 Baseline characteristics

Baseline characteristics were well-balanced between groups in the primary VA-ECMO analysis cohort (Table 2), with no significant differences in demographics, illness severity (APACHE-II scores), etiology of ECMO initiation, key comorbidities, or vital signs at ECMO initiation (all *p* > 0.05).

**Table 2:** Comparison of baseline characteristics between the two groups (*n* = 44).

Variables	Group R (*n* = 22)	Group M (*n* = 22)	*t/χ^2^* value	*p*-value
Age, years	51.91 ± 1.90	51.27 ± 2.05	1.067	0.292
Sex				
Male, *n* (%)	11 (50.0)	13 (59.1)	0.092	0.763
Female, *n* (%)	11 (50.0)	9 (40.9)		
BMI, kg/m^2^	24.60 ± 1.45	24.58 ± 1.65	0.039	0.969
Illness severity				
APACHE-II scores	20.59 ± 2.04	20.64 ± 1.81	−0.078	0.938
The etiology of ECMO initiation				
Cardiac arrest, *n* (%)	7 (31.8)	5(22.7)	0.115	0.735
Cardiogenic shock, *n* (%)	9 (40.9)	7 (31.8)	0.098	0.754
Respiratory failure, *n* (%)	6 (27.3)	10 (45.5)	0.884	0.347
Key comorbidities				
Hypertension, *n* (%)	12 (54.5)	9 (40.9)	0.364	0.546
Diabetes mellitus, *n* (%)	8 (36.4)	10 (45.5)	0.094	0.759
Coronary artery disease, *n* (%)	14 (63.6)	10 (45.5)	0.825	0.364
Vitals at ECMO initiation				
Heart rate, beats/min	104.91 ± 12.09	104.14 ± 10.72	0.224	0.824
Mean Arterial pressure, mmHg	66.32 ± 6.05	64.45 ± 4.54	1.155	0.255
Mechanically ventilated, *n* (%)	22(100)	22(100)	NA	NA

Data are presented as mean ± SD unless otherwise indicated.

Baseline laboratory values after ECMO cannulation are presented in Table 3. Both groups showed elevated white blood cell counts and decreased albumin levels, consistent with systemic inflammation and critical illness. All laboratory parameters, including coagulation markers, were comparable between Group R and Group M, with no statistically significant differences (all *p* > 0.05).

**Table 3:** Comparison of the baseline laboratory indicators between the two groups (*n* = 44).

Indicator	Group R (*n* = 22)	Group M (*n* = 22)	*t/Z* value	*p-value*
White blood cell count, 10^9^/L	10.66 (10.27–11.25)	10.30 (10.15–11.25)	−0.846	0.398
Platelet count, 10^9^/L	129 (126–135)	129 (126–132)	−0.130	0.897
Total bilirubin, μmol/L	11.7 ± 1.52	11.2 ± 2.20	0.868	0.391
Albumin, g/L	30.7 ± 4.55	30.3 ± 3.86	0.311	0.757
Serum creatinine, μmol/L	91.1 (84.8–95.3)	93.3 (83.0–95.2)	−0.094	0.925
PT, s	13.4 ± 1.50	13.3 ± 1.34	0.340	0.736
APTT, s	42.2 ± 1.95	41.4 ± 3.34	0.887	0.381
INR	1.15 (1.00–1.55)	1.30 (1.00–1.50)	−0.035	0.972

Data are presented as mean ± SD or median (IQR).

The exploratory VV-ECMO cohort (*n* = 8) showed similar distributions of age, sex, and illness severity between groups, with all patients receiving ECMO for respiratory failure. Data for this cohort are presented descriptively in Supplementary Tables S1, S2.

### 3.2 Primary outcome

For the primary outcome, delirium occurred in 0 of 22 patients (0%) in the remimazolam group compared to 6 of 22 patients (27.3%) in the midazolam group. The risk difference was −27.3% (95% CI −45.9% to −8.7%), corresponding to a number needed to treat (NNT) of 3.7 (95% CI 2.2 to 12.3). Fisher's exact test showed a statistically significant difference between groups (*p* = 0.021; Table 4).

**Table 4:** Comparison of the incidence of delirium between the two groups (*n* = 44).

Outcome	Group R (*n* = 22)	Group M (*n* = 22)	Effect estimate (95% CI)	*p*-*value*
Delirium events, *n* (%)	0 (0)	6 (27.3)	Risk difference: −27.3% (−45.9% to −8.7%)	0.021^a^
			NNT: 3.7 (2.2 to 12.3)	
CAM-ICU-assessable patient-days, days	3 (3 -4)	5 (4–5.75)	Median difference: −1.0 (−2.0 to 0.0)	0.025^b^
Delirium density, days/100 patient-days	0	23.53	Rate difference: −23.52 (−23.52 to −18.48)^c^	< 0.001^c^

^a^Fisher's exact test.

^b^Mann-Whitney U-test, data presented as median with interquartile range (IQR).

^c^Poisson exact test, due to zero events in Group R, the lower bound of the 95% CI for the rate difference equals the point estimate.

To assess robustness to potential unmeasured confounding, we calculated the E-value. The E-value for the point estimate was 2.0, meaning that an unmeasured confounder would need to be associated with both sedation choice and delirium by a risk ratio of at least 2.0-fold to fully explain away the observed protective effect. However, the E-value for the lower bound of the confidence interval was 1.3, indicating that even a relatively weak unmeasured confounder (risk ratio > 1.3) could shift the confidence interval to include the null. Consequently, the robustness of our findings to unmeasured confounding is limited, and residual confounding by indication remains a distinct possibility (Table 4).

#### 3.2.1 Assessment opportunity analysis

Because the remimazolam-based regimen maintained more stable sedation with less fluctuation, patients in Group R had fewer days with spontaneous RASS ≥ −3. The median number of CAM-ICU-assessable patient-days was 3 days (Q1 = 3, Q3 = 4) in Group R and 5 days (Q1 = 4, Q3 = 5.75) in Group M (*p* = 0.025).

#### 3.2.2 Assessment denominator-adjusted analysis

After adjustment using the assessment denominator approach, delirium density was 0.0 days per 100 patient-days in Group R compared to 23.53 days per 100 patient-days in Group M (rate difference: −23.53, 95% CI: −23.53 to −18.48), with a statistically significant difference by Poisson exact test (*p* < 0.001).

#### 3.2.3 Calendar period distribution

All patients in Group M (*n* = 22) were enrolled before June 2024, and all patients in Group R (*n* = 22) were enrolled after June 2024. The two groups were completely separated in calendar period with no overlap. This temporal trend itself is a potential source of bias; however, it is noteworthy that during this period, ECMO management protocols, intensive care practices, and delirium assessment procedures remained unchanged other than the choice of sedative.

#### 3.2.4 Sensitivity analysis (confounding by indication)

**Figure 2:**
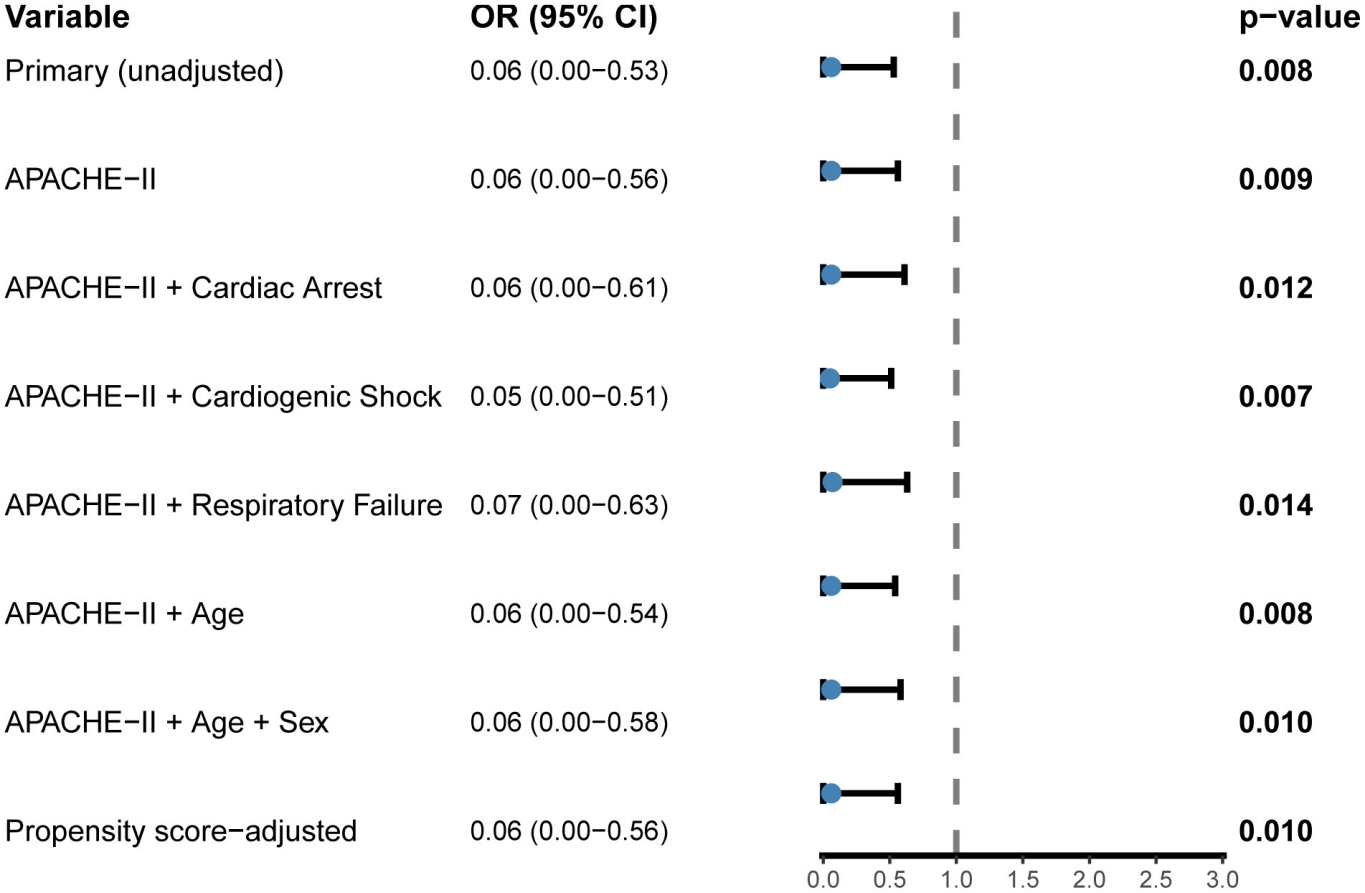
Forest plot of sensitivity analyses. Odds ratios (OR) and 95% confidence intervals for delirium with remimazolam-based vs. midazolam-based sedation regimens across different adjustment models are shown. “Primary (unadjusted)” represents the unadjusted model with only the group variable; other models represent stepwise addition of covariates to the primary model; “Propensity score (full model)” represents adjustment for the full propensity score incorporating all prespecified covariates (APACHE-II, age, sex, and etiology of ECMO initiation). Blue circles represent point estimates of OR, horizontal lines represent 95% confidence intervals, and the dashed vertical line indicates OR = 1 (null). All models yielded OR < 1 with 95% CIs excluding 1.

To control for confounding by indication, we performed multivariable-adjusted sensitivity analyses, with results presented as a forest plot (Figure 2). After adjusting for APACHE-II, the OR for delirium with the remimazolam-based regimen vs. the midazolam-based regimen was 0.06 (95% CI: 0.00–0.56, *p* = 0.009). After further adjusting for etiology for ECMO initiation (cardiac arrest, cardiogenic shock, respiratory failure), ORs ranged from 0.05 to 0.07, with all 95% CIs excluding 1. After propensity score adjustment, the OR was 0.06 (95% CI: 0.00–0.56, *p* = 0.010). While all sensitivity analyses yielded results consistent with the primary analysis (OR range 0.05–0.07), the E-value analysis (based on risk difference) yielded a point estimate of 2.0 with a lower CI bound of 1.3. This indicates that a relatively modest unmeasured confounder could potentially shift the confidence interval to the null, highlighting the exploratory nature of these findings and the possibility of residual confounding.

### 3.3 Secondary outcome

#### 3.3.1 Sedation-related indicators and adverse events

Patients in Group R achieved the sedation target more rapidly (10.37 ± 1.04 min vs. 12.39 ± 1.67 min, *p* < 0.001) and had significantly shorter recovery time after decannulation (24.90 ± 3.36 h vs. 29.84 ± 4.53 h, *p* < 0.001) compared to Group M. Muscle strength was better preserved in Group R [1 (1, 2) grades vs. 0 (0–1) grades, *p* < 0.001]. ICU length of stay was shorter in Group R (9.41 ± 1.47 d vs. 14.45 ± 2.52 d, *p* < 0.001), while total ECMO duration was similar between groups (7.41 ± 1.47 d vs. 7.45 ± 1.26 d, *p* = 0.913; Table 5).

**Table 5:** Comparison of sedation indicators and adverse events between the two groups (*n* = 44).

Outcome	Group R (*n* = 22)	Group M (*n* = 22)	*t/Z* value	*p*-value
Sedation indicators				
ECMO duration, days	7.41 ± 1.47	7.45 ± 1.26	−0.110	0.913
Muscle strength, grades	1 (1–2)	0 (0–1)	−3.581	< 0.001
Time to achieve sedation target, min	10.37 ± 1.02	12.39 ± 1.67	−4.824	< 0.001
Recovery time after decannulation, hours	24.90 ± 3.36	29.84 ± 4.53	−4.114	< 0.001
Time spent in the ICU, days	9.41 ± 1.47	14.45 ± 2.52	−8.111	< 0.001
Adverse events				
Hypotension, *n* (%)	1 (4.5)	8 (36.4)	–	0.021^a^
Bradycardia, *n* (%)	2 (9.1)	9 (40.9)	4.364	0.037

Data are presented as mean ± SD or median (IQR).

^a^Expected counts were less than 5, and comparisons between groups were made via Fisher's exact test.

Regarding adverse events, the incidences of hypotension (4.5% vs. 36.4%, *p* = 0.021) and bradycardia (9.1% vs. 40.9%, *p* = 0.037) were significantly lower in Group R compared with Group M (Table 5). Results for injection site pain, nausea, vomiting, and respiratory depression are presented in the Supplementary Table S6. Limitations regarding the measurement of these outcomes in deeply sedated patients are addressed in Section 4.

#### 3.3.2 Sedation quality

RASS scores over time are presented in Table 6. While no between-group difference existed at baseline (T0, *p* = 0.275), both groups underwent gradual lightening of sedation as clinical conditions improved. RASS scores progressively increased from approximately −4 at T1 to −1 or −2 at T7. Patients receiving the remimazolam regimen exhibited more stable sedation levels with less fluctuation throughout this period. The midazolam group showed greater variability with more frequent spontaneous lightening of sedation.

**Table 6:** Comparison of sedation quality between the two groups (*n* = 44).

Time	Group R (*n* = 22)	Group M (*n* = 22)	*Z value*	*p*-value
T0	−4.00 (−4.00 to −3.25)	−4.00 (−4.00 to −3.00)	−1.092	0.275
T1	−4.00 (−4.00 to −3.25)	−3.00 (−4.00 to −3.00)	−2.092	0.036
T2	−4.00 (−4.00 to −4.00)	−3.50 (−4.00 to −3.00)	−2.187	0.029
T3	−4.00 (−4.00 to −4.00)	−3.00 (−4.00 to −3.00)	−2.282	0.023
T4	−4.00 (−4.00 to −4.00)	−3.00 (−4.00 to −3.00)	−3.084	0.002
T5	−3.00 (−3.00 to −3.00)	−3.00 (−3.00 to −2.25)	−2.852	0.004
T6	−2.00 (−2.00 to −2.00)	−1.00 (−2.00 to −1.00)	−3.017	0.003
T7	−2.00 (−2.00 to −1.00)	−1.00 (−2.00 to 0.00)	−2.438	0.015

Data are presented as median (IQR).

#### 3.3.3 Co-management of drug doses

In the primary VA-ECMO analysis cohort, all patients received standardized adjunctive therapy with dexmedetomidine and remifentanil alongside the primary sedative (remimazolam or midazolam). To maintain the target sedation depth (RASS −4 to −3), the dosage of dexmedetomidine and remifentanil were lower in Group R compared to Group M. Propofol was administered as a low-dose rescue bolus (0.1–0.6 mg/kg) in 6 patients (27.3%) in Group R during invasive procedures. It was not used for continuous maintenance sedation in any patient. Propofol was not used in Group M.

Descriptive data for the 8 VV-ECMO patients are presented in Supplementary Tables S1–S5. Trends were generally consistent with the primary VA-ECMO findings, with lower delirium incidence and fewer adverse events observed in the remimazolam group.

## 4 Discussion

Optimal sedation management in patients receiving extracorporeal membrane oxygenation (ECMO) remains a significant clinical challenge. These patients require deep sedation to ensure patient-ventilator synchrony, reduce oxygen consumption, and prevent hemodynamic instability during the acute phase of critical illness (1, 16). However, prolonged exposure to sedative agents carries substantial risks, including delirium, ICU-acquired weakness (ICU-AW), and delayed recovery, all of which negatively impact long-term outcomes (17–19). While traditional sedatives such as midazolam and propofol are commonly used, their pharmacokinetic limitations—including active metabolite accumulation, hepatic metabolism dependence, and dose-dependent cardiopulmonary depression—may exacerbate these complications in ECMO patients (17, 19–21). Therefore, identifying sedative agents with more favorable pharmacokinetic and safety profiles is essential for improving outcomes in this vulnerable population. Injectable remimazolam benzenesulfonate, an ultrashort-acting benzodiazepine with organ-independent metabolism and rapid clearance, represents a promising alternative (7, 22–24). However, clinical evidence regarding its use in ECMO patients remains limited, and this study aims to address this gap by systematically comparing remimazolam-based vs. midazolam-based sedation regimens in this specific population.

Recent high quality systematic reviews and meta-analyses have reported that remimazolam is associated with a low overall incidence of postoperative delirium (approximately 5%) and can be safely used in surgical patients. Li et al. (8) analyzed 29 randomized controlled trials including 2,435 patients and found a pooled delirium incidence of 5% following remimazolam administration, with higher rates in high-risk subgroups such as ASA III IV patients (19%) and those undergoing orthopedic surgery (12%). Similarly, Mahendru et al. (9) compared remimazolam with inhalational anesthesia and highlighted its favorable safety profile in general surgical populations. However, these analyses predominantly included patients undergoing standard surgical procedures, and critically ill populations, particularly those requiring ECMO support, were systematically excluded. The pharmacokinetic profile of sedative agents can be significantly altered in ECMO patients due to hemodilution, drug sequestration within the circuit, and organ dysfunction (25), making extrapolation from general surgical populations potentially unreliable. To date, no study has specifically investigated remimazolam-based sedation in this unique population. This study addresses this critical evidence gap by providing the first exploratory data on remimazolam-based vs. midazolam-based sedation regimens in VA-ECMO patients.

### 4.1 Main finding

This retrospective cohort study provides the first exploratory data on remimazolam-based vs. midazolam-based sedation regimens in patients receiving VA-ECMO support. The core finding was a significantly lower incidence of delirium in the remimazolam-based regimen compared to the midazolam-based regimen (0% vs. 27.3%, *p* = 0.021). This corresponds to an absolute risk reduction of 27.3% and a number needed to treat of 3.7. The remimazolam-based regimen was also associated with favorable secondary outcomes, including shorter recovery time after decannulation, better-preserved muscle strength, faster achievement of sedation targets, and lower incidence of hypotension and bradycardia. While sensitivity analyses (including assessment-denominator and multivariable adjustments) yielded consistent results of the primary association, the E-value analysis suggested that residual confounding by indication remains a distinct possibility. In summary, these findings suggest that the remimazolam-based sedation regimen was associated with favorable outcomes in this exploratory cohort. However, these results should be strictly considered hypothesis-generating and require rigorous validation in prospective studies.

### 4.2 Potential impact of ascertainment bias

Delirium assessment in this study required a RASS score ≥ −3. As shown in Table 6, the remimazolam-based regimen maintained more stable sedation levels with less fluctuation as sedation was gradually lightened. In contrast, the midazolam group showed greater variability, with more frequent spontaneous lightening to RASS ≥ −3. Because of this difference in sedation stability, patients in the remimazolam group had fewer days with RASS ≥ −3 and therefore fewer CAM-ICU assessment opportunities. The median number of assessable patient-days was 3 days in Group R compared to 5 days in Group M (*p* = 0.025). This difference in assessment frequency could potentially introduce ascertainment bias.

After adjustment for differential assessment opportunities using delirium density (delirium-positive days per 100 assessable patient-days), the incidence of delirium remained significantly lower in Group R than in Group M (0.0 vs. 23.5 per 100 patient-days, *p* < 0.001). This finding indicates that the lower delirium rate cannot be fully explained by differences in assessment frequency alone. The more stable sedation profile of the remimazolam-based regimen may have contributed to this outcome. Nevertheless, although the delirium density analysis suggests that the difference in assessment frequency cannot fully account for the observed effect, it cannot entirely eliminate the possibility of an artifactual reduction in detected delirium. Direct comparison of raw delirium incidence between regimens with inherently different sedation stability profiles should be undertaken with caution. Future prospective studies employing standardized daily awakening protocols and systematic delirium screening are essential to definitively address this potential bias.

### 4.3 Clarification of regimen differences

It should be noted that the two groups differed not only in the primary sedative but also in the doses of adjunctive agents (dexmedetomidine, remifentanil). Propofol rescue was used only in the remimazolam group as a low-dose bolus during invasive procedures. It was not used for continuous maintenance sedation in any patient. Therefore, our findings reflect a comparison of two distinct sedation regimens rather than a head-to-head comparison of remimazolam and midazolam alone.

### 4.4 Relation with previous evidence

The findings of this exploratory study align with previous research and provide important additional real-world data regarding the clinical profile of a remimazolam-based sedation regimen, specifically addressing the evidence gap in ECMO patients identified by recent meta-analyses (8, 9). The relevance of our observed associations to existing evidence is analyzed below from several perspectives.

#### 4.4.1 Delirium incidence and clinical recovery

The observed lower delirium incidence in the remimazolam-based regimen represents an exploratory finding of this study. Delirium is a common and devastating complication in ICU patients, associated with prolonged mechanical ventilation, increased mortality, and long-term cognitive impairment (16, 26). While the absolute risk reduction of 27.3% (NNT = 3.7) was observed in our study, this effect size must be interpreted with caution due to the small sample size (*n* = 22 per group), which likely contributes to statistical instability rather than a definitive clinical effect. This is evidenced by the wide confidence intervals (OR 0.06, 95% CI 0.00–0.56) and a lower E-value bound of 1.3, indicating that residual confounding could move the interval to the null.

Crucially, this observed difference in delirium should be viewed as a regimen-based association rather than evidence of pure drug superiority. The remimazolam-based regimen maintained more stable sedation levels with less fluctuation. This stability resulted in fewer days with spontaneous RASS ≥ −3 and therefore fewer CAM-ICU assessment opportunities. This difference in assessment frequency introduces a substantial risk of ascertainment bias. Although we addressed this using an assessment-denominator approach (delirium density), an artifactual lowering of delirium incidence cannot be entirely excluded. Furthermore, the overall synergy of the regimen—including significantly lower doses of adjunct dexmedetomidine and remifentanil—may have synergistically contributed to these findings.

From a pharmacologic perspective, the clinical characteristics of the regimen may be supported by the unique properties of remimazolam. As an ultrashort-acting benzodiazepine, remimazolam is rapidly hydrolyzed to inactive metabolites by tissue esterases (22–24), which may prevent drug accumulation—a factor particularly relevant in ECMO patients with altered pharmacokinetics due to drug sequestration and organ dysfunction (25). In contrast, midazolam's hepatic metabolism to active metabolites can contribute to prolonged sedation, especially in patients with renal impairment (17, 19). While these pharmacokinetic profiles may facilitate a more controllable sedation regimen, these preliminary findings are strictly hypothesis-generating and require rigorous validation in prospective randomized trials.

#### 4.4.2 Comparison with traditional sedatives and drug combination strategies

The clinical observations in this exploratory cohort invite comparison with traditional sedation strategies. While propofol is commonly used during ECMO, its potential for dose-related cardiovascular and respiratory depression remains a concern (20, 21). In our study, the remimazolam-based regimen was associated with lower observed incidences of respiratory depression (4.5% vs. 31.8%) and hypotension (4.5% vs. 36.4%), Propofol was used only as a low-dose rescue bolus in a minority of patients and was not used for continuous sedation. However, these findings should be interpreted with caution given the retrospective nature of the study and the inherent difficulties in documenting these events in deeply sedated ECMO patients.

Dexmedetomidine is known for its neuroprotective effects but is associated with hypotension and bradycardia (27–29). In this study, both groups received dexmedetomidine as baseline sedation, providing a consistent context for comparison. Within this framework, the lower delirium incidence observed in the remimazolam-based regimen should not be attributed solely to the pharmacological properties of remimazolam itself. Instead, it likely reflects the overall synergy of the sedation regimen, which included significantly lower doses of adjunct dexmedetomidine and remifentanil in Group R, as well as the selective use of propofol rescue. Rather than demonstrating drug-specific superiority, our findings suggest that this specific combination regimen may represent a feasible and potentially efficient sedation strategy for this complex patient population, warranting further validation in prospective randomized trials.

#### 4.4.3 Sedation efficiency and clinical outcomes

In this exploratory analysis, the remimazolam-based regimen was associated with a significantly faster achievement of target sedation compared to the midazolam-based regimen. This faster onset is consistent with the pharmacokinetic profile of remimazolam as an ultrashort-acting benzodiazepine (22). Prompt control of agitation is clinically critical during the acute phase of ECMO support, as it helps prevent hemodynamic instability (16). Furthermore, RASS score trajectories showed that the remimazolam group maintained more stable sedation levels from 6 h through 9 days. This predictable and controllable sedation is likely attributed to remimazolam rapid metabolism by non-specific tissue esterases and the absence of active metabolites (23, 24).

Furthermore, the shorter recovery time after decannulation observed in the remimazolam cohort may facilitate earlier neurological assessment and mobilization (18), potentially contributing to the observed reduction in ICU length of stay. Nevertheless, given the small retrospective dataset and the differences in adjunct medication doses between groups, these findings should be interpreted as hypothesis-generating. The observed clinical improvements represent a regimen-based association rather than definitive drug-specific superiority, and larger prospective studies are necessary to validate these exploratory findings.

#### 4.4.4 Recovery profile and muscle strength

Beyond the lower delirium incidence, this study also highlights clinical recovery characteristics associated with the remimazolam-based regimen. Patients in the remimazolam group exhibited a shorter recovery time after decannulation and potentially better-preserved muscle strength. These observations are consistent with the rapid metabolic clearance of remimazolam and the absence of active metabolites (22, 24), which may facilitate earlier awakening and minimize cumulative sedative effects—crucial factors for early mobilization and rehabilitation in the ICU setting.

ICU-acquired weakness (ICU-AW) remains a critical factor affecting long-term survival and quality of life in patients receiving ECMO (18). The observed difference in muscle strength (median MRC grade 1 vs. 0) suggests that this specific sedation regimen may help mitigate the inhibitory effects on the neuromuscular junction that can be associated with the accumulation of certain sedative agents (30). However, it is important to note that these improvements represent a regimen-based association rather than drug-specific superiority. The lower doses of adjunct medications required in Group R may have also contributed to the more rapid recovery and better muscle strength preservation. Given the retrospective and exploratory nature of this study, these findings should be interpreted as hypothesis-generating, and prospective randomized trials are necessary to confirm whether these clinical recovery benefits are directly attributable to the choice of the primary sedative.

#### 4.4.5 Hemodynamic and respiratory safety

Regarding safety, our exploratory findings suggest an observed association between the remimazolam-based regimen and a more stable hemodynamic profile in this cohort. Traditional sedatives are often associated with risks of hypotension and bradycardia, particularly in hemodynamically unstable patients (20, 21). These results are consistent with observations from prior clinical trials (31, 32), suggesting that the remimazolam-based regimen may be associated with less pronounced hemodynamic depression, possibly due to its minimal effects on vascular tone and myocardial contractility (33). Such stability is particularly relevant for ECMO patients with limited cardiovascular reserve (1, 2).

However, these secondary safety endpoints must be interpreted with substantial qualification. The retrospective documentation of hypotension and bradycardia in complex ECMO patients carries a high risk of ascertainment bias, and the clinical validity of these measurements remains challenging to confirm. While the observed incidences of hypotension (4.5% vs. 36.4%), bradycardia (9.1% vs. 40.9%), and respiratory depression (4.5% vs. 31.8%) were lower in the remimazolam group, these findings are exploratory and should be viewed as regimen-based associations. The lower doses of adjunctive medications in Group R likely contributed to this observed stability. Given the inherent challenges in assessing these outcomes in deeply sedated, mechanically ventilated ECMO patients, our findings should be regarded as hypothesis-generating rather than definitive evidence of pharmacological superiority.

#### 4.4.6 Tolerability and other adverse reactions

As noted in our limitations, several secondary endpoints, including injection pain, nausea, and vomiting, are difficult to measure accurately in deeply sedated and mechanically ventilated ECMO patients. Therefore, these exploratory results must be interpreted with extreme caution. While we observed a lower incidence of injection pain in the remimazolam group (9.1% vs. 40.9%), which may be related to the formulation and reduced local stimulatory effect of remimazolam (34), these assessments were based on behavioral responses and patient recall, making them highly susceptible to documentation bias. Similarly, although the incidences of nausea (4.5% vs. 40.9%) and vomiting (4.5% vs. 31.8%) were recorded as lower in the remimazolam cohort, the clinical validity of these subjective outcomes in this specific population remains uncertain.

Both groups received dexmedetomidine as baseline adjuvant sedation. The observed benefits in Group R should be viewed as an association with the overall sedation regimen rather than being solely attributable to the pharmacokinetics of remimazolam. The lower required doses of adjunct dexmedetomidine and remifentanil in Group R, combined with the selective use of rescue propofol (27.3% in Group R vs. 0% routine use in Group M), likely contributed to the observed differences in tolerability and clinical efficiency. However, it is important to underscore that secondary safety endpoints, including injection site pain, nausea, vomiting, and respiratory depression, are notoriously difficult to measure accurately in deeply sedated, mechanically ventilated ECMO patients. Given the extremely high risk of documentation bias inherent in retrospective chart reviews, these specific endpoints should be regarded only as descriptive and supportive observations. We strongly discourage overinterpretation of these tolerability outcomes; their primary value lies in highlighting the need for standardized, systematic assessment protocols in future prospective studies. Rather than demonstrating drug-specific superiority, these findings suggest that the remimazolam-based combination regimen may offer a feasible and potentially more easily adjustable alternative for this complex patient population, though these observations require validation in prospective randomized trials.

### 4.5 Confounding by indication and treatment-era bias

Confounding by indication and treatment-era bias are inherent to our design. The transition to a remimazolam-based regimen in June 2024 resulted in a complete separation of calendar periods between the two groups. Although multivariable-adjusted and propensity score-adjusted sensitivity analyses yielded consistent results (OR range 0.05–0.07, Figure 2), residual confounding from unmeasured practice changes over time remains possible. Importantly, while these statistical adjustments were employed to mitigate treatment-era bias, they cannot fully eliminate the influence of unmeasured changes in overall patient care, ECMO management, or ICU protocols that may have co-occurred over the two calendar periods. These sensitivity analyses are therefore better viewed as partial mitigation rather than definitive control of treatment-era bias. Regarding statistical robustness, the lower confidence bound E-value of 1.3 indicates that a relatively modest unmeasured confounder could shift the observed association to the null. Therefore, the findings of this small exploratory study (*n* = 44) should not be interpreted as definitive evidence of superiority, but rather as hypothesis-generating observations that require validation in large-scale prospective randomized trials.

### 4.6 Strengths and limitations

This study has several strengths. First, it provides the first real-world evidence on remimazolam sedation specifically in ECMO patients, addressing a critical evidence gap highlighted by recent meta-analyses. Second, the study employed standardized sedation protocols with consistent adjuvant sedation in both groups, enhancing comparability. Third, we applied rigorous statistical methods including Firth regression, E-value analysis, assessment denominator adjustment, and multivariable sensitivity analyses to address analytical challenges.

Despite the significant associations observed, this study has several important limitations that merit careful consideration. First, as a single-center retrospective study with strict inclusion criteria (e.g., requiring at least 24 h of prespecified sedation and excluding early deaths within 48 h), there is an inherent risk of selection bias, as our findings may primarily apply to a more stable subpopulation surviving the initial hyper-acute phase of ECMO support. The observed associations may also reflect local practice patterns, including institutional sedation protocols, the specific ECMO circuit used, and multidisciplinary team dynamics, which may limit the generalizability of the findings to centers with different practices. Claims of generalizability beyond similar ECMO settings should therefore be avoided. Second, confounding by indication and treatment-era bias are highly probable because the sedative regimens were selected based on clinical judgment and institutional drug availability (with remimazolam introduction in mid-2024). Although we mitigated this through propensity-score matching and adjusting for prespecified covariates, the E-value analysis (lower CI bound of 1.3) indicates that a relatively modest unmeasured confounder could still shift the confidence interval to the null, meaning residual confounding cannot be entirely excluded, and these sensitivity analyses are best viewed as partial mitigation rather than definitive control of treatment-era bias. Third, the exposure contrast between the two groups was not strictly isolated. Both regimens incorporated dexmedetomidine and remifentanil at varying doses. Propofol rescue was used only in the remimazolam group as a low-dose procedural bolus and was not used for continuous maintenance sedation. This reflects a regimen-based association rather than a pure drug-specific superiority. Fourth, the modest sample size (*n* = 44) fundamentally limits our statistical power, leading to effect-size uncertainty and wide confidence intervals. This limited sample size directly contributes to the wide confidence intervals around our effect estimates, particularly the odds ratio for the primary outcome (OR 0.06, 95% CI 0.00–0.56). The effect sizes observed should therefore be regarded as unstable estimates intended to guide hypothesis generation rather than to inform clinical decision-making. Our findings should be strictly interpreted as exploratory hypotheses requiring prospective validation rather than definitive evidence of efficacy. Fifth, delirium assessment required RASS ≥ −3. The more stable sedation profile of the remimazolam-based regimen resulted in fewer assessable patient-days (median 3 vs. 5 days, *p* = 0.025). Although delirium density analysis reduced this ascertainment bias and the between-group difference remained significant (0.0 vs. 23.5 per 100 patient-days, *p* < 0.001), it does not eliminate the possibility that more stable deep sedation artifactually reduced delirium detection. Direct comparison of raw delirium incidence between regimens with inherently different sedation stability profiles should be undertaken with caution. Finally, secondary safety endpoints such as respiratory depression, injection site pain, nausea, and vomiting are notoriously difficult to measure accurately in deeply sedated, mechanically ventilated ECMO patients. Given the high risk of documentation bias inherent in retrospective chart reviews, these endpoints should be regarded only as descriptive and supportive observations, and overinterpretation should be strongly discouraged. We have therefore deprioritized these endpoints to the Supplementary material. Their primary value lies in highlighting the critical need for standardized, systematic assessment protocols in future prospective studies to isolate the specific clinical profile of the sedative regimen from the generalized effect of profound sedation.

## 5 Conclusion

In this retrospective exploratory study, a sedation strategy built around remimazolam was associated with lower delirium incidence, better muscle strength recovery, and shorter ICU length of stay compared with a strategy built around midazolam. However, the two regimens differed not only in the primary sedative but also in the doses of adjunctive agents and the selective use of rescue propofol in the remimazolam group. Consequently, these findings should not be interpreted as evidence of the pharmacological superiority of remimazolam as a single agent over midazolam, but rather as support for the feasibility and potential clinical advantages of this specific combination regimen. Although the use of low-dose propofol rescue in the remimazolam group represents a potential confounder, the overall trend toward these improved short-term clinical outcomes is noteworthy. It must be emphasized that these findings reflect an observed association between two distinct sedation regimens within a small cohort, rather than evidence of the pure superiority of remimazolam as a single agent. In summary, the observed associations pertain to the overall sedation strategy and not to the isolated pharmacodynamic effect of any single drug. These preliminary findings are strictly hypothesis-generating and require rigorous validation in larger, prospective randomized controlled trials.

## Data Availability

The original contributions presented in the study are included in the article/Supplementary material, further inquiries can be directed to the corresponding author.

## Supplementary material

Supplementary Table S1 Comparison of baseline characteristics between the two groups in the exploratory VV- ECMO analysis cohort.

Supplementary Table S2 Comparison of baseline laboratory indicators between the two groups in the exploratory VV-ECMO analysis cohort.

Supplementary Table S3 Comparison of sedation indicators between the two groups in the exploratory VV-ECMO analysis cohort.

Supplementary Table S4 Comparison of sedation quality between the two groups in the exploratory VV-ECMO analysis cohort.

Supplementary Table S5 Comparison of adverse events between the two groups in the exploratory VV-ECMO analysis cohort.

Supplementary Table S6 Comparison of other adverse events between the two groups in the VA-ECMO analysis cohort.

Supplementary Table S7 STROBE checklist.

Supplementary Data S1 Clinical dataset for the VA-ECMO cohort.

Supplementary Data S2 Clinical dataset for the VV-ECMO cohort.
