# Supplementary material for: Clinical outcomes of a remimazolam-based sedation regimen in patients receiving ECMO: a retrospective comparative study
Source: Front Med (Lausanne). 2026 Jun 8;13:1819593. doi: 10.3389/fmed.2026.1819593 (PMC13284138; doi:10.3389/fmed.2026.1819593)
Supplement: Supplementary Table S1 — Comparison of baseline characteristics between the two groups in the exploratory VV- ECMO analysis cohort. [file Table_1.docx]

**Table S1. Comparison of baseline characteristics between the two groups in the exploratory VV-ECMO analysis cohort (n=8)**

| Project | Group R (n =4) | Group M (n = 4) |
| --- | --- | --- |
| Age, years | 51.50 (50.75 - 52.00) | 51.50 (50.75 - 52.25) |
| sex |  |  |
| Male, n (%) | 3 (75.0) | 2 (50.0) |
| Female, n (%) | 1 (25.0) | 2 (50.0) |
| BMI, kg/m^2^ | 24.55 (23.96 - 25.00) | 24.27 (23.70 - 24.93) |
| Illness Severity |  |  |
| APACHE-Ⅱ scores | 20.50 (19.75 - 21.25) | 21.00 (20.75 - 21.25) |
| The etiology of ECMO initiation |  |  |
| Respiratory failure. n (%) | 4 (100) | 4 (100) |
| Key comorbidities |  |  |
| Hypertension, n (%) | 2 (50) | 1 (25) |
| Diabetes mellitus, n (%) | 1 (25) | 2 (50) |
| Coronary artery disease, n (%) | 1 (25) | 2 (50) |
| Vitals at ECMO initiation |  |  |
| Heart rate, beats/min | 101.5 (99.8 -103.5) | 102.5 (99.3 - 104.5) |
| Mean Arterial pressure, mmHg | 62.0 (59.3 - 65.0) | 61.0 (59.8 - 62.5) |
| Mechanically Ventilated, n (%) | 4 (100) | 4 (100%) |
