# Supplementary material for: Clinical outcomes of a remimazolam-based sedation regimen in patients receiving ECMO: a retrospective comparative study
Source: Front Med (Lausanne). 2026 Jun 8;13:1819593. doi: 10.3389/fmed.2026.1819593 (PMC13284138; doi:10.3389/fmed.2026.1819593)
Supplement: Supplementary Table S2 — Comparison of baseline laboratory indicators between the two groups in the exploratory VV-ECMO analysis cohort. [file Table_2.docx]

**Table S2. Comparison of baseline laboratory indicators between the two groups in the exploratory VV-ECMO analysis cohort (n=8)**

| Indicator | Group R (n=4) | Group M (n=4) |
| --- | --- | --- |
| White blood cell count, 10^9^/L | 11.87 (10.93 - 12.68) | 11.39 (11.17 - 11.73) |
| Platelet count, 10^9^/L | 142 (138 - 146) | 139 (138 - 141) |
| Total bilirubin, μmol/L | 11.3 (10.0 - 13.0) | 12.1 (11.0 - 13.1) |
| Albumin, g/L | 29.8 (27.6 - 33.0) | 30.6 (29.5 - 31.6) |
| Serum creatinine, μmol/L | 82.50 (77.78 - 87.50) | 81.30 (79.88 - 82.58) |
| PT, s | 13.3 (12.8 - 14.1) | 13.7 (13.2 - 13.9) |
| APTT, s | 43.3 (42.0 - 44.6) | 41.6 (40.5 - 42.6) |
| INR | 1.20 (1.15 - 1.23) | 1.15 (1.08 - 1.23) |
