# Supplementary material for: Clinical outcomes of a remimazolam-based sedation regimen in patients receiving ECMO: a retrospective comparative study
Source: Front Med (Lausanne). 2026 Jun 8;13:1819593. doi: 10.3389/fmed.2026.1819593 (PMC13284138; doi:10.3389/fmed.2026.1819593)
Supplement: Supplementary Table S3 — Comparison of sedation indicators between the two groups in the exploratory VV-ECMO analysis cohort. [file Table_3.docx]

**Table S3. Comparison of sedation indicators between the two groups in the exploratory VV-ECMO analysis cohort (n=8)**

| Indicators | Group R (n=4) | Group M (n=4) |
| --- | --- | --- |
| Total ECMO Operating Time, days | 12.50 (11.50 - 13.25) | 16.50 (15.5 - 16.50) |
| Muscle Strength, grade | 1.5 (1.0 - 2.0) | 0.5 (0 - 1.0) |
| Time to Achieve Sedation Target, min | 14.98 (14.47 - 15.48) | 24.83 (23.13 - 25.56) |
| Recovery Time after Deactivation, hours | 12.00 (11.85 - 12.15) | 25.00 (22.17 - 26.63) |
| Time Spent in the ICU, days | 14.50 (13.50 - 15.25) | 18.50 (17.50 - 19.25) |
| CAM-ICU-assessable patient-days, days | 8.0 (7.5 - 9.0) | 14.5 (13.8 - 15.5) |
| Delirium density, days/100 patient-days | 0 | 25.42 |
