# Supplementary material for: Clinical outcomes of a remimazolam-based sedation regimen in patients receiving ECMO: a retrospective comparative study
Source: Front Med (Lausanne). 2026 Jun 8;13:1819593. doi: 10.3389/fmed.2026.1819593 (PMC13284138; doi:10.3389/fmed.2026.1819593)
Supplement: Supplementary Table S4 — Comparison of sedation quality between the two groups in the exploratory VV-ECMO analysis cohort. [file Table_4.docx]

**Table S4. Comparison of sedation quality between the two groups in the exploratory VV-ECMO analysis cohort (n = 8)**

| Time | Group R (n=4) | Group M (n=4) |
| --- | --- | --- |
| T0 | -3.50 (-4.00 - -3.00) | -3.50 (-4.00 - -3.00) |
| T1 | -3.50 (-4.25 - -3.00) | -3.00 (-3.25 - -3.00) |
| T2 | -4.00 (-4.25 - -4.00) | -4.00 (-4.00 - -3.75) |
| T3 | -3.50 (-4.25 - -3.00) | -3.00 (-3.25 - -3.00) |
| T4 | -4.00 (-4.00 - -3.75) | -3.00 (-3.25 - -3.00) |
| T5 | -3.00 (-3.25 - -3.00) | -3.00 (-3.00 - -2.75) |
| T6 | -2.50 (-3.00 - -2.00) | -2.00 (-2.25 - -2.00) |
| T7 | -2.00 (-2.00 - -1.75) | -1.00 (-1.25 - -1.00) |
