# Supplementary material for: Clinical outcomes of a remimazolam-based sedation regimen in patients receiving ECMO: a retrospective comparative study
Source: Front Med (Lausanne). 2026 Jun 8;13:1819593. doi: 10.3389/fmed.2026.1819593 (PMC13284138; doi:10.3389/fmed.2026.1819593)
Supplement: Supplementary Table S5 — Comparison of adverse events between the two groups in the exploratory VV-ECMO analysis cohort. [file Table_5.docx]

**Table S5. Comparison of adverse events between the two groups in the exploratory VA-ECMO analysis cohort (n=8)**

| Adverse reaction | Group R (n=4) | Group M (n=4) |
| --- | --- | --- |
| Hypotension, n (%) | 1 (25.0) | 3 (75.0) |
| Bradycardia, n (%) | 1 (25.0) | 3(75.0) |
| Respiratory depression, n (%) | 0 (0) | 3 (75.0) |
| Nausea, n (%) | 0 (0) | 2 (50.0) |
| Vomiting, n (%) | 1 (25.0) | 3 (75.0) |
| Injection site pain, n (%) | 0 (0) | 3 (75.0) |
| Delirium, n (%) | 0 (0) | 3 (75.0) |
