# Supplementary material for: Clinical outcomes of a remimazolam-based sedation regimen in patients receiving ECMO: a retrospective comparative study
Source: Front Med (Lausanne). 2026 Jun 8;13:1819593. doi: 10.3389/fmed.2026.1819593 (PMC13284138; doi:10.3389/fmed.2026.1819593)
Supplement: Supplementary Table S6 — Comparison of other adverse events between the two groups in the VA-ECMO analysis cohort. [file Table_6.docx]

**Table S6 Comparison of other adverse events between the two groups in the VA-ECMO analysis cohort (n = 44)**

| Adverse reaction | Group R (n=22) | Group M (n=22) | *χ^2^* value | *p* value |
| --- | --- | --- | --- | --- |
| Respiratory depression, n (%) | 1 (4.5) | 7 (31.8) | - | 0.046^a^ |
| Nausea, n (%) | 1 (4.5) | 9 (40.9) | 6.341 | 0.012 |
| Vomiting, n (%) | 1 (4.5) | 7 (31.8) | - | 0.046^a^ |
| Injection site pain, n (%) | 2 (9.1) | 9 (40.9) | 4.364 | 0.037 |

Data are presented as n (%).

^a^ Expected counts were less than 5, and comparisons between groups were made via Fisher's exact test.
