# Supplementary material for: Clinical outcomes of a remimazolam-based sedation regimen in patients receiving ECMO: a retrospective comparative study
Source: Front Med (Lausanne). 2026 Jun 8;13:1819593. doi: 10.3389/fmed.2026.1819593 (PMC13284138; doi:10.3389/fmed.2026.1819593)
Supplement: Supplementary Table S7 — STROBE checklist. [file Table_7.pdf]

STROBE Statement—Checklist of items that should be included in reports of ***cohort studies***

	Item No	Recommendation	Location in manuscript (Page & Line)	Reported
**Title and abstract**	1	(*a*) Indicate the study's design with a commonly used term in the title or the abstract	Page 1， Title	Yes
		(*b*) Provide in the abstract an informative and balanced summary of what was done and what was found	Page 1-2, Abstract, Line 14-39	Yes
**Introduction**				
Background/rationale	2	Explain the scientific background and rationale for the investigation being reported	Page 2, Introduction, Line 41-64	Yes
	e	State specific objectives, including any prespecified hypotheses	Page 2, Introduction, Line 65-74	Yes
**Methods**				
Study design	4	Present key elements of study design early in the paper	Page 3, Methods, Line 77-80	Yes
Setting	5	Describe the setting, locations, and relevant dates, including periods of recruitment, exposure, follow-up, and data collection	Page 3, Methods, Line 81-86	Yes
Participants	6	(*a*) Give the eligibility criteria, and the sources and methods of selection of participants. Describe methods of follow-up	Page 3, Methods, Line 90-100	Yes
		(*b*) For matched studies, give matching criteria and number of exposed and unexposed	NA	No
Variables	7	Clearly define all outcomes, exposures, predictors, potential confounders, and effect modifiers. Give diagnostic criteria, if applicable	Page 4-5, methods, line 143-179	Yes
Data sources/ measurement	8*	For each variable of interest, give sources of data and details of methods of assessment (measurement). Describe comparability of assessment methods if there is more than one group	Page 4-5, Methods, Line 135-141	Yes
Bias	9	Describe any efforts to address potential sources of bias	Page 6, Methods, Line 198-222	Yes
Study size	10	Explain how the study size was arrived at	Page 3, Methods, Line 77-86	Yes
Quantitative variables	11	Explain how quantitative variables were handled in the analyses. If applicable, describe which groupings were chosen and why	Page 3, Methods, Line 77-86	Yes
Statistical methods	12	(*a*) Describe all statistical methods, including those used to control for confounding	Page 5-6, Methods, Line 180-230	Yes
		(*b*) Describe any methods used to examine subgroups and interactions	Page 5, Method, Line 180-196	

		(*c*) Explain how missing data were addressed	Page 6, Methods, Line 229-230	Yes
		(*d*) If applicable, explain how loss to follow-up was addressed	NA	Yes
		(*e*) Describe any sensitivity analyses	Page 5-6, Methods, Line 197-222	Yes
**Results**				
Participants	13*	(a) Report numbers of individuals at each stage of study—eg numbers potentially eligible, examined for eligibility, confirmed eligible, included in the study, completing follow-up, and analysed	Page 3, Methods, Line 81-86	Yes
		(b) Give reasons for non-participation at each stage	NA	No
		(c) Consider use of a flow diagram	Page 3, Methods, Line 86	Yes
Descriptive data	14*	(a) Give characteristics of study participants (eg demographic, clinical, social) and information on exposures and potential confounders	Page 6-7, Results, Line 233-244	Yes
		(b) Indicate number of participants with missing data for each variable of interest	NA	No
		(c) Summarise follow-up time (eg, average and total amount)	NA	No
Outcome data	15*	Report numbers of outcome events or summary measures over time	Page 7-8, Results, Line 246-315	Yes
Main results	16	(*a*) Give unadjusted estimates and, if applicable, confounder-adjusted estimates and their precision (eg, 95% confidence interval). Make clear which confounders were adjusted for and why they were included	Page 7-8, Results, Line 246-285	Yes
		(*b*) Report category boundaries when continuous variables were categorized	Page 7, Results, Line 246-257	Yes
		(*c*) If relevant, consider translating estimates of relative risk into absolute risk for a meaningful time period	Page 7-8, Results, Line 258-285	Yes
Other analyses	17	Report other analyses done—eg analyses of subgroups and interactions, and sensitivity analyses	Page 8, Results, Line 286-315	Yes
**Discussion**				
Key results	18	Summarise key results with reference to study objectives	Page 9, Discussion, Line 348-360	Yes
Limitations	19	Discuss limitations of the study, taking into account sources of potential bias or imprecision. Discuss both direction and magnitude of any potential bias	Page 13-14, Discussion, Line 531-577	Yes
Interpretation	20	Give a cautious overall interpretation of results considering objectives, limitations, multiplicity of	Page 10-13, Discussion, Line 361-530	Yes

		analyses, results from similar studies, and other relevant evidence		
Generalisability	21	Discuss the generalisability (external validity) of the study results	Page 15, Conclusion, Line 579-593	Yes
**Other information**				
Funding	22	Give the source of funding and the role of the funders for the present study and, if applicable, for the original study on which the present article is based	Page 15, Funding, Line 600-602	Yes

*Give information separately for exposed and unexposed groups.

Note: An Explanation and Elaboration article discusses each checklist item and gives methodological background and published examples of transparent reporting. The STROBE checklist is best used in conjunction with this article (freely available on the Web sites of PLoS Medicine at http://www.plosmedicine.org/, Annals of Internal Medicine at http://www.annals.org/, and Epidemiology at http://www.epidem.com/). Information on the STROBE Initiative is available at http://www.strobe-statement.org.
